# Climate change mitigation in Canada’s forest sector: a spatially explicit case study for two regions

**DOI:** 10.1186/s13021-018-0099-z

**Published:** 2018-09-06

**Authors:** C. E. Smyth, B. P. Smiley, M. Magnan, R. Birdsey, A. J. Dugan, M. Olguin, V. S. Mascorro, W. A. Kurz

**Affiliations:** 1 Natural Resources Canada, Canadian Forest Service, 506 Burnside Road West, Victoria, BC V8Z 1M5 Canada; 2 USDA Forest Service and Woods Hole Research Center, 149 Woods Hole Road, Falmouth, MA 02540 USA; 3 USDA Forest Service, Northern Research Station, 11 Campus Blvd, Suite 200, Newtown Square, PA 19073 USA; 4 Commission for Environmental Cooperation, 393 St-Jacques Street West, Suite 200, Montreal, QC H2Y 1N9 Canada

**Keywords:** Climate change mitigation scenario, Forest sector, CBM-CFS3, CBMF-HWP, Spatially explicit, Displacement factor

## Abstract

**Background:**

We determine the potential of forests and the forest sector to mitigate greenhouse gas (GHG) emissions by changes in management practices and wood use for two regions within Canada’s managed forest from 2018 to 2050. Our modeling frameworks include the Carbon Budget Model of the Canadian Forest Sector, a framework for harvested wood products that estimates emissions based on product half-life decay times, and an account of marginal emission substitution benefits from the changes in use of wood products and bioenergy. Using a spatially explicit forest inventory with 16 ha pixels, we examine mitigation scenarios relating to forest management and wood use: increased harvesting efficiency; residue management for bioenergy; reduced harvest; reduced slashburning, and more longer-lived wood products. The primary reason for the spatially explicit approach at this coarse resolution was to estimate transportation distances associated with delivering harvest residues for heat and/or electricity production for local communities.

**Results:**

Results demonstrated large differences among alternative scenarios, and from alternative assumptions about substitution benefits for fossil fuel-based energy and products which changed scenario rankings. Combining forest management activities with a wood-use scenario that generated more longer-lived products had the highest mitigation potential.

**Conclusions:**

The use of harvest residues to meet local energy demands in place of burning fossil fuels was found to be an effective scenario to reduce GHG emissions, along with scenarios that increased the utilization level for harvest, and increased the longevity of wood products. Substitution benefits from avoiding fossil fuels or emissions-intensive products were dependent on local circumstances for energy demand and fuel mix, and the assumed wood use for products. As projected future demand for biomass use in national GHG mitigation strategies could exceed sustainable biomass supply, analyses such as this can help identify biomass sources that achieve the greatest mitigation benefits.

**Electronic supplementary material:**

The online version of this article (10.1186/s13021-018-0099-z) contains supplementary material, which is available to authorized users.

## Background

Forest sector mitigation can be achieved through activities that increase forest area, increase stand- and landscape-level carbon (C) density through forest management activities or conservation [1], and through the use of harvested wood products to store C and displace other greenhouse gas (GHG) emissions-intensive materials such as concrete, steel, plastics, and fossil fuels [2–6]. Our objective was to examine the climate change mitigation potential of a suite of mitigation activities for two forest management units in Canada. This work was part of a coordinated tri-national study which used a harmonized modeling approach for six regions in Canada, the United States ([7] in review) and Mexico [8].

Climate change mitigation was defined as the potential for GHG emission reductions or removal increases relative to a baseline without mitigation actions. Our analysis included forest management scenarios that (i) maintained or increased stand-level C density through a reduction in harvest levels, and (ii) managed harvest residues to reduce slashburning, or used residues to create energy and solid wood products to displace the use of fossil-based energy sources or emissions-intensive products. The analysis also included a scenario that shifted the commodity mix of harvested wood products (HWP) towards longer-lived products.

We build upon previous research which addressed mitigation scenarios at the national level in Canada [5, 9], at the state level for Mexico [8], at the provincial level for British Columbia [6]. However, in those spatially referenced analyses we were unable to identify community-level relevant options or estimate transportation distances associated with collection of harvest residue for bioenergy. In this analysis, the objective was to rank the impacts of climate change mitigation activities for two regions using spatially explicit forest inventories, harvest projections and road networks. We refined energy substitution impacts by using the cheapest transportation routes to determine harvest residue availability for each community and by using community-level baseline data on energy and the associated emissions profile. In GHG analyses such as this one, transportation distances are rarely included, or short haul transportation distances are assumed [10–13]. However, we include transportation distances because the location of the woody biomass feedstock relative to the nearest conversion facility plays a pivotal role in the economic feasibility of bioenergy production [14–17]. The primary reason for the spatially explicit approach at the coarse resolution considered in these case studies was to better estimate the transportation distance, and other spatial aspects are not considered here.

Global change impacts on forest growth, decomposition, or disturbance regimes were not included in either the baseline or the mitigation scenarios. We did not examine deforestation or afforestation scenarios. There may be mitigation possibilities through avoided deforestation, but at the national level only ~ 0.02% of the forest area is annually affected by deforestation in Canada [18, 19].

## Methods

### Analytical frameworks

Our analysis considered mitigation potential to be reduced GHG emissions or enhanced C sequestration that would result from implementation of a mitigation option, relative to a baseline (IPCC, 2014). This approach assesses the potential climate change mitigation resulting from changes in forest management, the use of longer-lived products or bioenergy, and substitution impacts. We assume that all factors such as the potential impact of a changing climate or altered disturbance regimes have similar impacts on the baseline and a mitigation scenario, and thus do not contribute to changes in rankings of mitigation options. We defined forest sector mitigation based on C stock changes in the forest ecosystem and in HWPs. We used the IPCC Production Approach for estimating HWP C balances [20], following Canada’s approach for international reporting [18]. Under this approach we track C in wood that is harvested in Canada, regardless of where in the world these products are used.

Forest ecosystem C dynamics in this study were estimated using the Carbon Budget Model of the Canadian Forest Sector (CBM-CFS3) [21, 22]. Carbon transferred from forest ecosystems to HWP and bioenergy were tracked through manufacturing, use/export, and end-of-life use by the Carbon Budget Modeling Framework for Harvested Wood Products (CBM-FHWP) [5, 18]. More details about the models are described in Additional file 1: Additional materials.

Our analysis was conducted at a spatial resolution of 16 ha, for two Forest Management Units (FMUs) identified in Canada’s 2016 National GHG Inventory Report [23]. An FMU is an administrative unit, based on a designated area established by a provincial or territorial government, for which harvesting activities are permitted for approved forest management plans. Annual allowable cut levels are defined for these regions, which specify the maximum amount of timber that can be harvested on a sustainable basis for each management area. Modeling assumptions for each FMU were based on a forecast of future harvest volumes and wildfires. Harvest projections were provided by provincial experts, and annual future wildfire was assumed to be the same as the historical (1990–2014) average annual area burned, which in both FMUs is very low (1.1 kha/year in Cranbrook, 520 ha/year in Dog River).

### Study regions

**Fig. 1:**
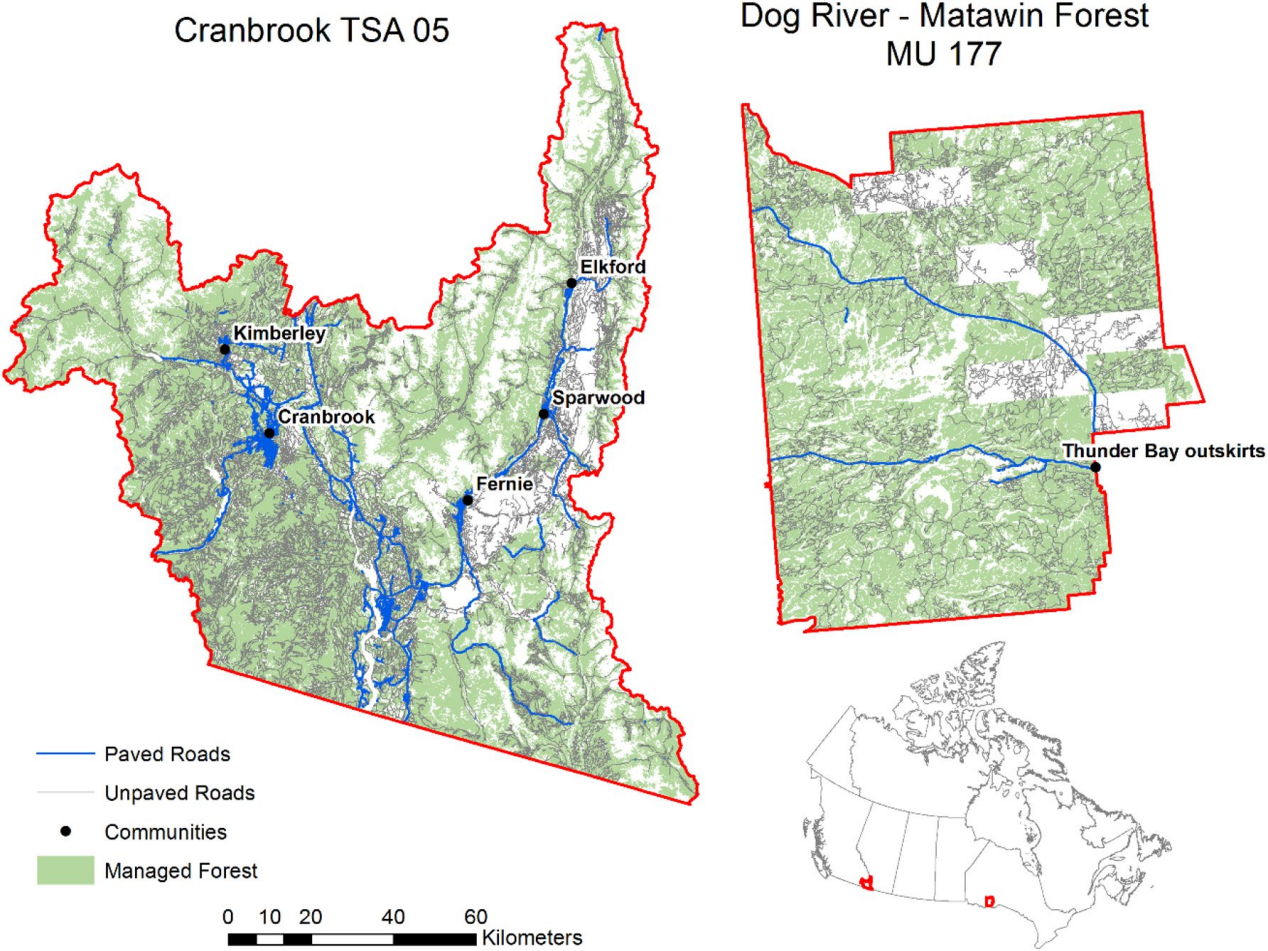
Maps of study areas for managed public forests within Cranbrook, British Columbia (Timber Supply Area 05) and Dog River-Matawin, Ontario (Management Unit 177), and the locations of these Forest Management Units within Canada (inset)

The two study regions were Cranbrook, British Columbia (BC) and Dog River-Matawin Forest in Ontario (ON), Fig. 1. Both regions are roughly 1 Mha in size, mainly coniferous species with spatially explicit forest attributes (species, age, silvicultural intensity or site class) and associated merchantable yield tables. Table 1 contains information on the forest inventory, anthropogenic and natural disturbances, and baseline energy usage. Transportation distances from harvest cutblocks to communities were estimated from networks of paved roads and unpaved forestry roads. If more than one community was present, the cheapest route from cutblock to community was selected assuming travel costs on paved roads were half the cost of travel on all other road types [24]. Road networks were analyzed using ESRI Network Analyst (v 10.3.1) [25].

**Table 1:** Study region characteristics, forest inventory information and baseline assumptions for Cranbrook (BC) and Dog River (ON)

Category	Description	Cranbrook (BC)	Dog River (ON)
Climate	Mean annual air temperature	1.5 °C	0.8 °C
Forest Inventory	Total area	1 Mha	0.75 Mha
	Management Unit	Timber supply area 05	4 W 177
	Baseline year	2011	2010
	Leading species	Lodgepole pine, Douglas fir, fir, spruce	Black spruce, poplar, jack pine, white birch
	# Records (400 m^2^, 16 ha pixels)	59.1 k	46.2 k
	Merchantable yield tables	Gross merchantable volume (VDYP7) based on site index and classifiers	Gross merchantable volume (Mist 3)
	Classifiers	Montane Cordillera ecozone, leading species, ownership, harvest eligibility, growth curve key, pixel X ID, pixel Y ID	Boreal Shield West ecozone, species mix, Forest Unit, silvicultural intensity, harvest eligibility, pixel X ID, pixel Y ID
Projected Activity Data	Harvest amount	1,292,000 m^3^/year in 2020 decreasing to 1,101,000 m^3^/year in 2040	513,000 m^3^/year in 2020 decreasing to 409,000 m^3^/year in 2040
	Harvest methods	85% utilization rate, minimum 60 year age, eligible stands sorted by highest merchantable C, slashburn 50% of harvested area	90% utilization rate, minimum 60 year age, eligible stands sorted by highest merchantable C, capture 10% of roundwood for bioenergy, 25% of residues for bioenergy and slashburn 25% of harvested area
	Wildfire	1.1 kha/year	0.5 kha/year
	Land use change	None	None
Harvested Wood Products	Bioenergy from roundwood	Bioenergy from roundwood 0%	Bioenergy from roundwood 10%
		Milling efficiency 76% of roundwood used for commodities	Milling efficiency 76% of roundwood used for commodities
	Mill residues	Mill residue used for bioenergy 30% capture	Mill residue used for bioenergy 15% capture
	Commodity proportions	Sawnwood 42%	Sawnwood 50.4%
		Panels 16.2%	Panels 19.5%
		Other solid wood 3.6%	Other solid wood 4.3%
		Pulp and paper 38.2%	Pulp and paper 25.6%
Infrastructure	Road layers (Accessed March 10, 2017)	GeoBC Atlas: Integrated Transportation Network, Government of BC, 2016	National Road Network, Natural Resources Canada, 2012
		Forestry tenure road segment lines, Government of BC, 2016	MNRF Road Network, Government of Ontario, 2016

### Substitution impacts

Two substitution impacts were included: marginal substitution between solid wood products (sawnwood and panels) and emissions-intensive materials, and marginal substitution between bioenergy from harvest residues and fossil fuel based stationary combustion for power and heat. Displacement factors for solid wood products (DFp) (sawnwood and panels) were previously estimated for Canada assuming wood substituted for emissions-intensive products within a series of end-use products (e.g. single-family homes, furniture, etc.) and partitioning total avoided emissions into sawnwood and panels according to their share in total consumption. Displacement factors consider emissions associated with extraction, transportation of raw materials and manufacturing. Two sets of displacement factors were analyzed. The first set, based on a broad range of end-use products, was 0.54 and 0.45 tC emissions avoided per tC used for sawnwood and panels, respectively [26]. The second set of displacement factors assumed the wood products were used for building construction and substituted for steel and concrete, resulting in higher displacement factors of 2.1 tC/tC and 2.2 tC/tC for sawnwood and panels, respectively [6]. These displacement factors included the production stage (extraction, transportation of raw materials, and manufacturing) and quantified from the change in emissions (for a set of more-wood products versus less-wood products) divided by the marginal change in wood C. Avoided emissions were estimated for a basket of end-use products and were weighted by Canadian consumption statistics to reflect national wood uses [26].

For avoided emissions for bioenergy, energy displacement factors (DFe) were estimated using a linear programming (LP) model that maximized avoided emissions by selecting from nine different candidate bioenergy facilities (Additional file 1: Table S2) to substitute for the most emissions-intensive baseline electricity and heat fuel sources [26]. In the first case, we assumed the energy demand was estimated by local population multiplied by a per capita energy use (Additional file 1: Table S1a), and assuming the fuel mix was the same as that used in the Province [26]. In the second case, we refined the displacement factors by including community-level energy demand and fuel mix in the BC case study [27], and by transporting residues across the FMU boundary to the adjacent community of Thunder Bay in the ON case study (Additional file 1: Table S1b). We assume that new bioenergy facilities would be constructed, and we do not include emissions associated with facility construction because we assume fossil energy sources would have similar construction or renovation emissions.

### Mitigation scenarios

Nine different individual and combination scenarios were assessed relative to the baseline, Table 2. The *Harvest Less* scenario reduced the harvest area 2–5 percentage points, which reduced the total amount of C transferred out of the ecosystem and the subsequent emissions from harvested wood products. However, fewer available wood products also reduces the substitution benefits. The *Higher Utilization* scenario kept the harvest area unchanged but increased the average harvest utilization rate by 5% which increased the harvest volume per hectare and reduced the amount of harvest residues and their related emissions from decay and/or slashburning. The incremental harvest volume was assumed to be usable for the same mix of commodities as the original harvest. The *Harvest Residues for Bioenergy* scenario maintained the harvest level and utilization rate of the baseline scenario, but reduced slashburning, and a portion of harvest residues (including branches, small trees, unused merchantable-sized trees and snags) was collected and transported to hypothetical bioenergy facilities to produce power and heat in place of using fossil fuels. The *No Slashburning* scenario, implemented in Ontario only, stopped slashburning activities in the mitigation scenario, leaving residues to decay. In the baseline, a portion of harvest residues was slashburned, causing immediate emissions of both carbon dioxide but also more potent GHGs (methane and nitrous oxide) from the burning. The longer-lived products (LLP) scenario shifted 4% of the wood fibre use from pulp and paper to panels which extended the retention period of C in HWPs and accrued substitution benefits from the incremental production of panels. The *LLP* scenario was combined with all forest management scenarios, discussed above, to quantify forest sector mitigation benefits.

**Table 2:** Individual mitigation scenario and description of activities

Scenario	Description	Parameter changed	Parameter value
Harvest less	Reduce harvest area	Harvest area	− 2% (BC) − 5% (ON)
Higher utilization	Increase the percentage of stemwood transferred to products	Harvest utilization rate	+ 5%
Harvest residues for bioenergy	Increase collection of harvest residues for bioenergy. Residues would otherwise decompose on forest floor or be slashburned	Slashburn area (percentage of harvest area) Harvest residue capture rate	− 25% + 25%
No slashburning	Stop slashburning activities in ON	Slashburn area (proportion of harvest area)	− 25%
Longer-lived products (LLP)	Increase the proportion of panels produced and reduce pulp and paper production.	HWP commodity percentage	+ 4%

Scenario combinations (not shown) were created by aggregating individual activities

## Results

### Energy displacement factors

Energy displacement factors were first estimated assuming provincial average fuel mix and energy consumption (Table 3). The displacement factor for Cranbrook was relatively high (0.95) because of the population of 47 k people and their associated electricity and fossil-intensive heat demand. In contrast, the small population within the Dog River FMU had a low energy demand, and most of the harvest residues were converted to electricity that was used to displace low emissions grid electricity (Table 4, Additional file 1: Table S3), resulting in a low displacement factor of 0.38.

**Table 3:** Collected harvest residues for bioenergy, energy demand and displacement factors for FMUs (forest management unit)

FMU	Population	Electricity demand (GWh)	Heat demand (GWh)	Residues (kodt)	Selected facilities	Displacement factor (tC/tC)
Cranbrook FMU, BC	47,232	1310.0	2620.1	161.5	13 CHP, 10 H	0.95
Dog River FMU, ON	532	6.9	13.8	31.5	1 CHP, 1 H, 18 E	0.38

*H* heat, *E* electricity, *CHP* combined heat and power, *odt* oven dry tonnes

**Table 4:** Avoided fossil fuels for the harvest residues for bioenergy scenario, where bioenergy facilities were selected to maximize avoided emissions

Region	Heat produced (GWh)	Electricity produced (GWh)	Electricity exported (GWh)	H: NG (GWh)	H: electricity (GWh)	H: fuel oil (GWh)	H: propane (GWh)	H: wood (GWh)	H: coke and petcoke (GWh)
Dog River, Ontario	11.3	4.5	26.3	6.4	0.6	0.7	0	0	3.6
Cranbrook FMU, BC	611.8	164.9	0	234.8	0	245.6	0	0	131.4
Thunder Bay, Ontario	116.2	34.3	0	0	0	116.2	0	0	0
Cranbrook	274.0	47.6	0	260.0	0	4.9	8.6	0.4	0
Elkford	40.8	0.7	0	35.7	0	1.8	3.2	0	0
Fernie	99.0	46.6	27.7	93.8	0	1.4	2.5	1.3	0
Kimberley	101.8	1.8	0	90.9	0	4.0	7.0	0	0
Sparwood	13.0	0.3	0	8.0	0	1.8	3.2	0	0

**Fig. 2:**
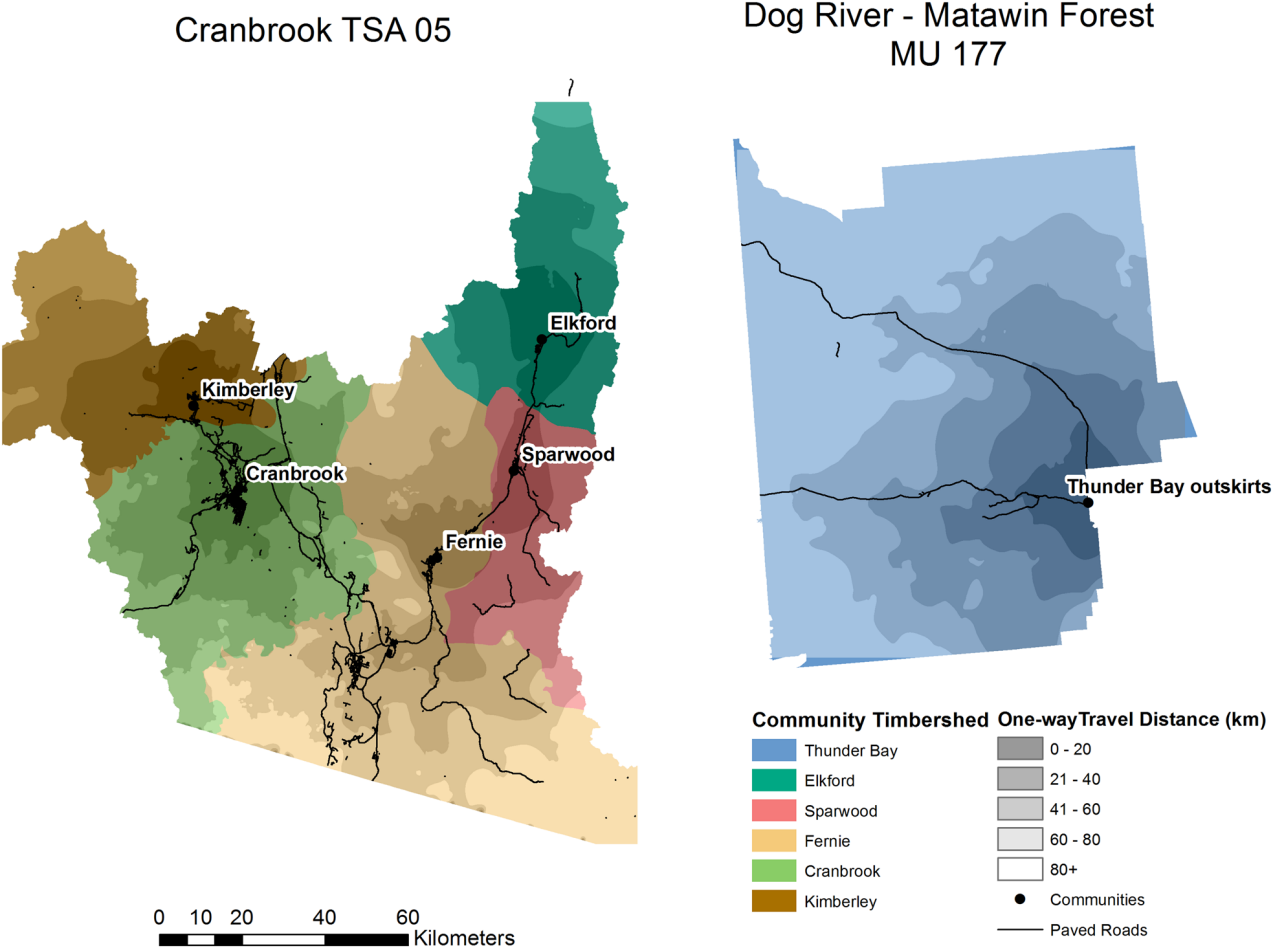
Map of transportation distances for cutblocks in Cranbrook and Dog River

The refined set of displacement factors considered the five communities within the Cranbrook FMU and their share of harvest residues based on cheapest transportation routes (Table 5). Energy demands were lower than the FMU average, and the average displacement factor was found to have a lower average value of 0.46 for the five communities, with a range of 0.23–0.66. For Dog River, transporting harvest residues to the nearby larger community of Thunder Bay (population 130 k) significantly increased the displacement factor from 0.38 to 1.0 because of the increased heat demand in that community and substitution of fuel oil. Average one-way transportation distances ranged from 31 to 65 km for the communities in the BC case study, and 76 km for Thunder Bay (Fig. 2).

**Table 5:** Collected harvest residues for bioenergy, energy demand, transportation distances and displacement factors for community-level

Communities	Northing (°)	Westing (°)	Population^a^	Electricity demand (GWh)	Heat demand (GWh)	Residues (kodt)	Selected facilities	Average transportation distance ± standard deviation (km) and [% paved]	Displacement factor (tC/tC)
Cranbrook	49.51549	115.7589	19,613	292.8	5 CHP, 6H	67.7	165.0	39 ± 16; [54%]	0.57
Kimberley	49.6871	115.9829	6576	130.6	1 CHP, 5H	22.1	54.9	31 ± 14; [14%]	0.66
Fernie	49.50676	115.0688	4479	103.4	1 CHP, 2H, 5E	60.4	46.6	65 ± 25; [65%]	0.23
Sparwood	49.73262	114.8919	3804	73.6	4H	3.5	32.1	41 ± 17; [60%]	0.58
Elkford	50.01225	114.9303	2520	50.6	3H	9.4	20.0	31 ± 16; [18%]	0.62
Thunder Bay (outskirts)	48.6748	89.8933	129,561	1678.7	3357.4	31.5	5 CHP, 1H	76 ± 28 [70%]	1.0

^a^Note that not all of the population is within the five communities

### Climate change mitigation potential

**Fig. 3:**
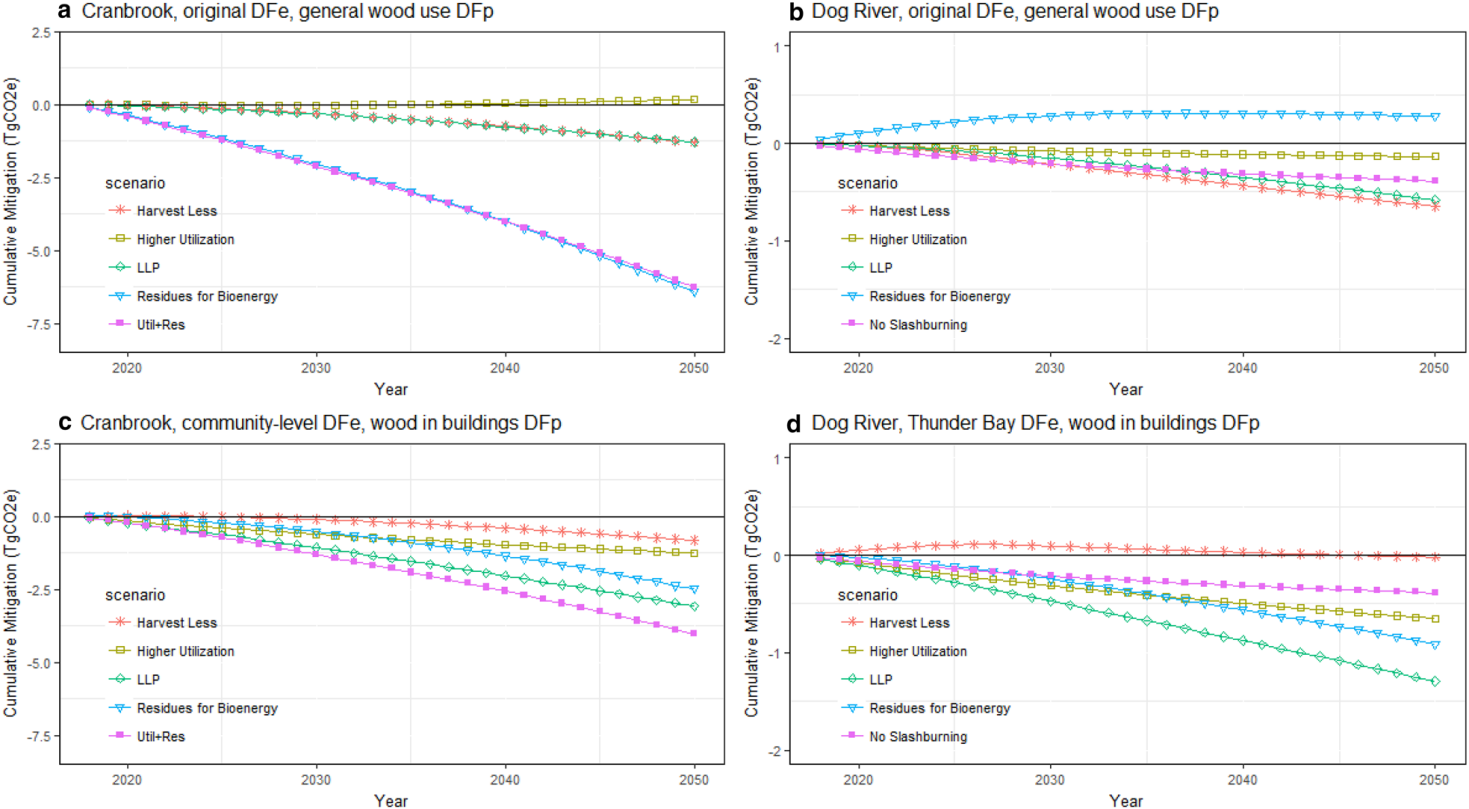
Cumulative mitigation for Cranbrook and Dog River FMUs with (**a**, **b**) displacement factors (DF) based on FMU-level energy substitution (DFe) and broad end-uses for solid wood products (DFp) or (**c**, **d**) displacement factors based on community-level energy substitution and incremental solid wood products for use in building construction. Negative values indicate a reduction in cumulative emissions. Abbreviations: LLP *Longer Lived Products,* Util. + Res. *Higher Utilization combined with Harvest Residues for Bioenergy*

**Fig. 4:**
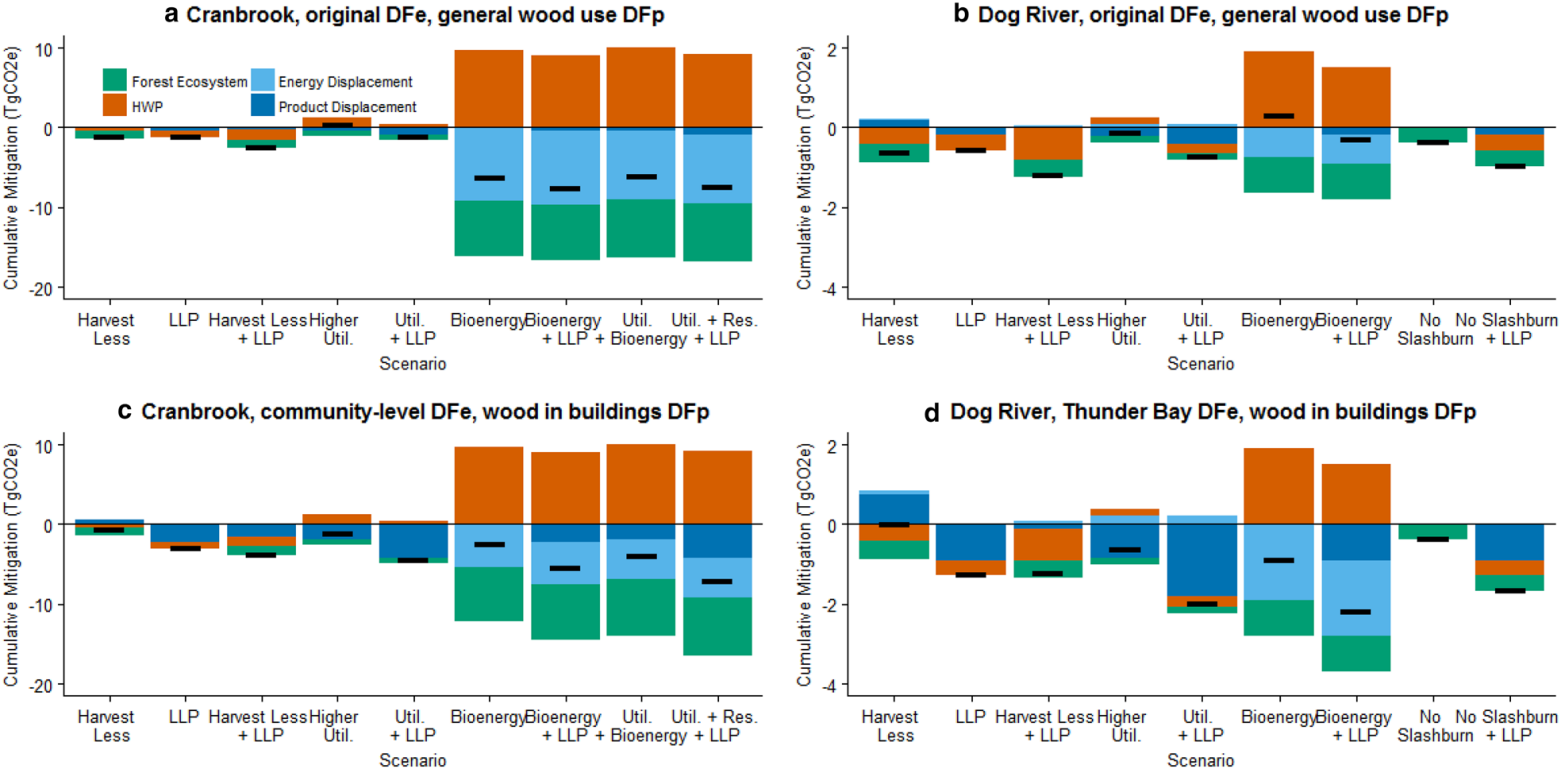
Total and component cumulative mitigation in 2050 for Cranbrook and Dog River FMUs with (**a**, **b**) displacement factors (DF) based on FMU-level energy substitution (DFe) and broad end-uses for solid wood products (DFp) or (**c**, **d**) displacement factors based on community-level energy substitution and incremental solid wood products for use in building construction. The black horizontal line shows the total mitigation. *LLP* longer lived products, *Util.* higher utilization, *Bioenergy or Res*. harvest residues for bioenergy

Time series of the mitigation potential showed that ranking of the activities could change over time, and that substitution benefits had considerable impact on the 2050 ranking (Fig. 3, see Additional file 1: Figure S3 in Additional materials for 2030 rankings). For all scenarios involving changes in forest management activity, the emissions were reduced relative to the baseline because of reduced emissions from decay and slashburning (*Higher Utilization, Residues for Bioenergy, No Slashburning*) or enhanced removals and reduced emissions related to lower harvest levels (*Harvest Less*). Harvesting transfers C to HWP, which temporarily stores and then emits C at the end of the products’ lifetime through incineration and landfill decay. Higher HWP emissions were associated with scenarios that used more wood products (*Higher Utilization*), while reduced emissions were associated with the *Harvest Less* scenario (Fig. 4). Substitution benefits depend on the marginal change in avoided emissions, which is based on the change in the amount of wood commodities, and the displacement factors. In the *Harvest Less* scenario the reduction in commodities accrued fewer substitution benefits, relative to the baseline, more so when displacement factors were high, which offset the emissions reductions in the forest and HWP components.

The *Higher Utilization* scenario had enhanced removals in the forest ecosystem because of reductions in slashburning and lower in situ decay relative to the baseline. Increased emissions associated with HWPs were offset by substitution benefits when displacement factors were high. The *Harvest Residues for Bioenergy* scenario had reduced emissions when substitution impacts were large, corresponding to situations where bioenergy was generated from heat to combined heat and power facilities and displaced fossil fuels. Stopping the burning of residues in the *No* *Slashburning* scenario reduced emissions because the baseline slashburning emitted C much faster than was the case when the C was left to decay in situ and did not generate non-CO_2_ emissions.

The *LLP* scenario had reduced emissions because longer product lifetimes delayed end-of-life emissions from HWPs relative to the baseline, and because of increased substitution benefits. Implementing two or more activities simultaneously in an FMU was found to achieve more mitigation than having only one individual scenario (Table 6).

**Table 6:** Average annual mitigation potential in (GgCO_2_e/year) for each decade, ranked by highest cumulative impact in 2050

Cranbrook				Dog River			
Scenario	2021–2030	2031–2040	2041–2050	Scenario	2021–2030	2031–2040	2041–2050
Higher utilization + harvest residues for bioenergy + LLP	− 197	− 228	− 257	Harvest residues for bioenergy + LLP	− 59	− 72	− 76
Harvest residues for bioenergy + LLP	− 134	− 180	− 217	Higher utilization + LLP	− 62	− 61	− 60
Higher utilization + harvest residues for bioenergy	− 108	− 126	− 147	No slashburning + LLP	− 52	− 51	− 49
Harvest less +LLP	− 95	− 124	− 143	Harvest less + LLP	− 31	− 45	− 45
Higher utilization +LLP	− 135	− 137	− 138	LLP	− 37	− 40	− 42
Harvest residues for bioenergy	− 50	− 84	− 114	Harvest residues for bioenergy	− 22	− 32	− 35
LLP	− 84	− 96	− 104	Higher utilization	− 23	− 18	− 16
Harvest less	− 12	− 30	− 42	No slashburning	− 15	− 10	− 7
Higher utilization	− 46	− 35	− 28	Harvest less	4	− 6	− 5

Higher product displacement factors and refined energy displacement factors have been used for each of the scenarios and combinations shown

## Discussion

These results did not include economic considerations, which could reduce the mitigation potential [28] but do include estimates of transportation distances for the bioenergy scenario. Although beyond the scope of the current analyses, economic and socio-economic analyses should also be addressed to understand potential barriers to implementation [6, 29]. Mitigation scenarios levels were considered to be feasible, but there are uncertainties about technical feasibility, regulatory barriers, and market barriers that were not considered. Forests provide a range of services and co-benefits, and forest managers are required to manage for multiple objectives, some of which could come into conflict with mitigation objectives and could limit the level of mitigation scenario implementation [30]. The mitigation scenarios considered in this analysis focused on harvesting activities, residue management and wood use, and did not consider activities which would impact growth and yield. Additional activities could be undertaken to enhance future growth which would enhance the forest sequestration potential [5, 28]. The *Higher Utilization* scenario assumed that the same product mix could be achieved with the 5% incremental utilization rate, i.e. incremental harvest from a stand, although the incremental harvest could be of lower quality and produce more short-lived wood commodities. On the other hand, technological advances in building materials such as cross-laminated timber and other composite materials such as oriented strand board are enabling higher utilization rates.

The *Harvest Less* mitigation potential was limited because reduced emissions from harvesting less were offset over time by a lower C uptake rate due to less post-harvest regeneration and fewer substitution benefits. Therefore, this scenario highlighted the trade-off between maintaining or increasing C density and increasing the C uptake rate by fulfilling societal demand for biomass [28]. We assumed that the small reduction in the production of pulp and paper would not have displacement impacts because consumers chose to use less of these products. We also assumed sawnwood and panels would be replaced by emissions-intensive products, rather than result in changes in harvesting and use of wood products from another region. Finally, we assumed that stands which were not harvested in this strategy would not be impacted by natural disturbances which could reverse the mitigation potential. Simulations with increased disturbance risk have found the increased burned area had negligible impacts on the mitigation potential [5, 31] because harvest targets were applied to larger geographic regions where the model could shift harvest to other eligible stands and future harvests could be achieved regardless of increased future area burned. This conclusion is of course highly dependent on the fraction of Annual Allowable Cut that is actually harvested and the magnitude of increases in natural disturbances [32–35]. Issues of leakage and risk of reversal of mitigation benefits (i.e. non-permanence) have been identified in earlier studies, but their impacts are difficult to project because these impacts are not usually included in mitigation analyses; in a review Buchholz et al. [36] found only 8 of 149 cases included leakage assessment, and non-permanence has more often been addressed in afforestation or reforestation projects [37].

The only scenario regarding wood use in this study, the *LLP* scenario, increased the C storage in HWP by transferring C from pulp and paper products (2 year half-life) to panels (25 year half-life) (IPCC, 2006). Similar to other studies, mitigation benefits were found to be further enhanced via displacement effects [38].

Comparing the two regions, there are differences in the mitigation potentials because harvest levels are different for the two regions, and the baseline parameters, scenario and scenario implementation levels also vary by region. For the *LLP* scenario, the mitigation potential was higher in Cranbrook because this region had much higher harvest levels and produced more structural wood products. Similarly, the *Higher Utilization* was higher for Cranbrook because of the higher harvest levels, and because the 5% increase in utilization was proportionally larger—the baseline utilization rate for Cranbrook assumed 85% of C in merchantable-sized trees was transferred to products in Cranbrook versus 90% in Dog River. The *Harvest Less* scenario had small mitigation benefits for both regions, with some differences between the two regions. Dog River had a higher implementation level for this scenario (5% reduction in harvest versus 2% reduction in Cranbrook), and a higher proportion of structural wood products (74.4% versus 61.8% in Cranbrook), which resulted in differences in the mitigation potential for the two regions (Fig. 4).

The trends for the *Harvest Residues for Bioenergy* scenario are consistent with previous findings, where the GHG benefit has been found to depend on the emissions intensity of the fossil fuel displaced [10, 39–41], as well as the conversion efficiency of the woody biomass combustion [42], and whether or not residues would have been slashburned or left to decay. In Dog River, which has less slashburning, the mitigation potential was driven more by the avoided fossil fuel emissions, particularly when residues were used in the nearby community of Thunder Bay. For the Cranbrook region, a greater enhancement was found in the forest component because of higher slashburning rates. The use of community-level data improved the quantification of substitution benefits by refining the displacement factors for specific timbershed regions. Future work could include the production of transportation biofuels for communities such as Fernie BC where stationary combustion had small substitution benefits. When the *Harvest Residues for Bioenergy* scenario was combined with the *Higher Utilization* scenario, fewer residues were available for bioenergy because they were used for solid wood products. Using the residues for products instead of bioenergy in the combination strategy *Higher Utilization *+* Harvest Residues for Bioenergy* resulted in higher mitigation potential when the additional commodities were used in buildings to displace steel and concrete.

## Conclusions

Canada’s forests and forest products can contribute to mitigating climate change, and various mitigation options are available for forest management and wood-product use, with very large differences in mitigation benefits among the scenarios. The study includes impacts of mitigation activities on carbon stocks and fluxes in forest ecosystems, harvested wood products, and changes in emissions resulting from use of wood products instead of other emissions-intensive products and energy sources. Cumulative changes to 2050 relative to a baseline scenario are quantified.

The use of harvest residues for local production of bioenergy was found to be effective, and we have included transportation distances associated with the transport of residue to nearby communities. A scenario that increased the harvest utilization level of merchantable-sized trees was also successful, and both of these scenarios included benefits from substituting for fossil fuels or emissions-intensive products. Combining forest management activities, such as higher utilization or bioenergy from harvest residues, with a wood-use scenario which generated more longer-lived products scenario had the highest mitigation potential. Refinements to substitution benefits have been included in this analysis, but there is still uncertainty in the substitution benefits and future research could focus on future policy directions (e.g. more use of wood in commercial and tall buildings, phase out of fuel oil for heating, reduced slash-pile burning, etc.). As projected future demand for biomass use in national GHG mitigation strategies could exceed sustainable biomass supply, analyses such as this can help identify biomass sources that achieve the greatest mitigation benefits.

## Additional file

**Additional file 1.** Additional materials.

## References

[1] Nabuurs GJ, Masera O, Andrasko K, Benitez-Ponce P, Boer R, Dutschke M (2007). IPCC forestry.

[2] Sathre R, Gustavsson L, Bergh J (2010). Primary energy and greenhouse gas implications of increasing biomass production through forest fertilization. Biomass Bioenergy.

[3] Werner F, Taverna R, Hofer P, Thürig E, Kaufmann E (2010). National and global greenhouse gas dynamics of different forest management and wood use scenarios: a model-based assessment. Environ Sci Policy.

[4] Obersteiner M, Böttcher H, Yamagata Y (2010). Terrestrial ecosystem management for climate change mitigation. Curr Opin Environ Sustain..

[5] Smyth CE, Stinson G, Neilson E, Lemprière TC, Hafer M, Rampley GJ (2014). Quantifying the biophysical climate change mitigation potential of Canada’s forest sector. Biogeosciences.

[6] Xu Z, Smyth CE, Lemprière TC, Rampley GJ, Kurz WA (2017). Climate change mitigation strategies in the forest sector: biophysical impacts and economic implications in British Columbia, Canada. Mitig Adapt Strateg Glob Change..

[7] Dugan AJ, Birdsey R, Mascorro VS, Magnan M, Smyth CE, Kurz WA, et al. Integrated modeling and assessment of climate change mitigation options in the united states forest sector. Carbon Balance Manag. 2018. In review. 10.1186/s13021-018-0100-x PMC6123328 30182168

[8] Olguin M, Wayson C, Fellows M, Birdsey R, Smyth CE, Magnan M (2018). Applying a systems approach to assess carbon emission reductions from climate change mitigation in Mexico’s forest sector. Environ Res Lett.

[9] Smyth C, Kurz WA, Rampley GJ, Lemprière TC, Schwab O (2017). Climate change mitigation potential of local use of harvest residues for bioenergy in Canada. Glob Change Biol Bioenergy..

[10] Laganière J, Paré D, Thiffault E, Bernier PY (2017). Range and uncertainties in estimating delays in greenhouse gas mitigation potential of forest bioenergy sourced from Canadian forests. GCB Bioenergy..

[11] Thakur A, Canter CE, Kumar A (2014). Life-cycle energy and emission analysis of power generation from forest biomass. Appl Energy.

[12] Jones G, Loeffler D, Calkin D, Chung W (2010). Forest treatment residues for thermal energy compared with disposal by onsite burning: emissions and energy return. Biomass Bioenergy.

[13] Domke GM, Becker DR, D’Amato AW, Ek AR, Woodall CW (2012). Carbon emissions associated with the procurement and utilization of forest harvest residues for energy, northern Minnesota, USA. Biomass Bioenergy..

[14] Nepal S, Contreras MA, Lhotka JM, Stainback GA (2014). A spatially explicit model to identify suitable sites to establish dedicated woody energy crops. Biomass Bioenergy.

[15] Hellmann F, Verburg PH (2011). Spatially explicit modelling of biofuel crops in Europe. Biomass Bioenergy.

[16] Lundmark R, Athanassiadis D, Wetterlund E (2015). Supply assessment of forest biomass—a bottom-up approach for Sweden. Biomass Bioenergy.

[17] Shabani N, Akhtari S, Sowlati T (2013). Value chain optimization of forest biomass for bioenergy production: a review. Renew Sustain Energy Rev.

[18] Environment and Climate Change Canada (2017). National Inventory Report: 1990–2015, greenhouse gas sources and sinks in Canada Ottawa.

[19] Kurz WA, Shaw CH, Boisvenue C, Stinson G, Metsaranta J, Leckie D (2013). Carbon in Canada’s boreal forest—a synthesis. Environ Rev..

[20] IPCC (2013). Revised supplementary methods and good practice guidance arising from the Kyoto Protocol.

[21] Kurz WA, Dymond CC, White TM, Stinson G, Shaw CH, Rampley GJ (2009). CBM-CFS3: a model of carbon-dynamics in forestry and land-use change implementing IPCC standards. Ecol Model.

[22] Metsaranta JM, Shaw CH, Kurz WA, Boisvenue C, Morken S (2017). Uncertainty of inventory-based estimates of the carbon dynamics of Canada’s managed forest (1990–2014). Can J For Res.

[23] Environment and Climate Change Canada (2016). National Inventory Report: 1990–2014, greenhouse gas sources and sinks in Canada.

[24] Ralevic P (2013). Evaluating the greenhouse gas mitigation potential and cost-competitiveness of forest bioenergy systems in Northeastern Ontario.

[25] ESRI (2015). Esri ArcGIS 10.3.1.

[26] Smyth CE, Rampley GJ, Lemprière TC, Schwab O, Kurz WA (2017). Estimating product and energy substitution benefits in national-scale mitigation analyses for Canada. Glob Change Biol Bioenergy..

[27] Community Energy and Emissions Inventory. 2016. http://www2.gov.bc.ca/gov/content/environment/climate-change/data/ceei. Accessed 2 Nov 2016.

[28] Lemprière TC, Kurz WA, Hogg EH, Schmoll C, Rampley GJ, Yemshanov D (2013). Canadian boreal forests and climate change mitigation. Environ Rev.

[29] Lemprière TC, Krcmar E, Rampley GJ, Smyth CE, Hafer M (2017). The cost of climate change mitigation in Canada’s forest sector. Can J For Res.

[30] Golden D, Smith MA, Colombo S (2011). Forest carbon management and carbon trading: a review of Canadian forest options for climate change mitigation. For Chron..

[31] Metsaranta JM, Kurz WA, Neilson ET, Stinson G (2010). Implications of future disturbance regimes on the carbon balance of Canada’s managed forest (2010–2100). Tellus B..

[32] Kurz WA, Dymond CC, Stinson G, Rampley GJ, Neilson ET, Carroll AL (2008). Mountain pine beetle and forest carbon feedback to climate change. Nature.

[33] Balshi MS, McGuire AD, Duffy P, Flannigan M, Kicklighter DW, Melillo J (2009). Vulnerability of carbon storage in North American boreal forests to wildfires during the 21st century. Glob Change Biol..

[34] Weed AS, Ayres MP, Hicke JA (2013). Consequences of climate change for biotic disturbances in North American forests. Ecol Monogr.

[35] Price DT, Alfaro R, Brown K, Flannigan M, Fleming R, Hogg E (2013). Anticipating the consequences of climate change for Canada’s boreal forest ecosystems. Environ Rev..

[36] Buchholz T, Hurteau M, Gunn J, Saah D (2016). A global meta-analysis of forest bioenergy greenhouse gas emission accounting studies. GCB Bioenergy..

[37] Galik CS, Murray BC, Mitchell S, Cottle P (2016). Alternative approaches for addressing non-permanence in carbon projects: an application to afforestation and reforestation under the Clean Development Mechanism. Mitig Adapt Strateg Global Change..

[38] Gustavsson L, Haus S, Lundblad M, Lundström A, Ortiz CA, Sathre R (2017). Climate change effects of forestry and substitution of carbon-intensive materials and fossil fuels. Renew Sustain Energy Rev.

[39] Zanchi G, Pena N, Bird N (2012). Is woody bioenergy carbon neutral? A comparative assessment of emissions from consumption of woody bioenergy and fossil fuel. Glob Change Biol Bioenergy..

[40] Cintas O, Berndes G, Cowie AL, Egnell G, Holmström H, Ågren GI (2016). The climate effect of increased forest bioenergy use in Sweden: evaluation at different spatial and temporal scales. Wiley Interdiscip Rev Energy Environ..

[41] Guest G, Cherubini F, Strømman AH (2013). The role of forest residues in the accounting for the global warming potential of bioenergy. GCB Bioenergy..

[42] Richter D, Jenkins DH, Karakash JT, Knight J, McCreery LR, Nemestothy KP (2009). Wood energy in America. Science.

