# Supplementary material for: Climate change mitigation in Canada’s forest sector: a spatially explicit case study for two regions
Source: Carbon Balance Manag. 2018 Sep 6;13:11. doi: 10.1186/s13021-018-0099-z (PMC6125263; doi:10.1186/s13021-018-0099-z)
Supplement: Supplementary file 1 — Additional file 1. Additional materials. [file 13021_2018_99_MOESM1_ESM.docx]

**Supplementary Online Materials**

# S1. Forest ecosystem model

The greenhouse gas (GHG) emissions and removals in the forest ecosystem resulting from mitigation strategies described in this study were estimated using the Carbon Budget Model for the Canadian Forest Sector (CBM-CFS3) (1). The model was originally developed in the late 1980s (2) and is the same model used to produce forest ecosystem estimates for reporting in Canada’s annual GHG National Inventory Report (3, 4), and has been used in many other countries (e.g. 5, 6).

The CBM-CFS3 simulates annual carbon (C) transfers associated with ecosystem processes and natural and anthropogenic disturbances between the atmosphere and 10 biomass pools and 11 dead organic matter pools in the forest ecosystem. The model integrates forest inventory data, growth and yield data, and information on forest management practices and natural disturbance impacts. Annual ecosystem processes comprise growth, litter fall, mortality and decomposition and are simulated as C transfers executed annually in every inventory record. During the annual processes, C is taken up in the biomass pool and some biomass C is transferred to dead organic matter (DOM) pools (Figure S1).

Growth is simulated as an annual process. Every record in the forest inventory is associated with a yield table that defines the dynamics of merchantable volume over time. Assignment of an inventory record to the appropriate curve is based on a classifier set that includes administrative unit, ecological stratum, leading species, site productivity class and several other classifiers.

Conversion of merchantable volume curves to above-ground biomass curves is performed with a set of equations developed for Canada’s National Forest Inventory (7). These equations derive the above-ground biomass of each stand component from merchantable stemwood volume (per ha), for each province/territory, ecozone, leading species or forest type. Finally, root biomass pools are estimated from above-ground biomass pools using regression equations (8).

Rates of C transfer are defined for each pool, based on pool-specific turnover rates (for biomass pools) or decay rates. Turnover rates can be very high (e.g. 95% for hardwood foliage) or very low (e.g. < 1% for stemwood). The decay of DOM C results in its transfer to a slow soil pool or to the atmosphere. Annual decay rates are defined for a reference mean annual temperature of 10°C and exhibit temperature sensitivity according to defined Q10 relationships. The decay rates vary between 50% (very fast DOM pools receiving input from fine root turnover) and 0.0032% (slow soil pool representing mineral soil). Several studies have compared model predictions to measurements (9-11), and assessed sensitivity and uncertainty (12-15).


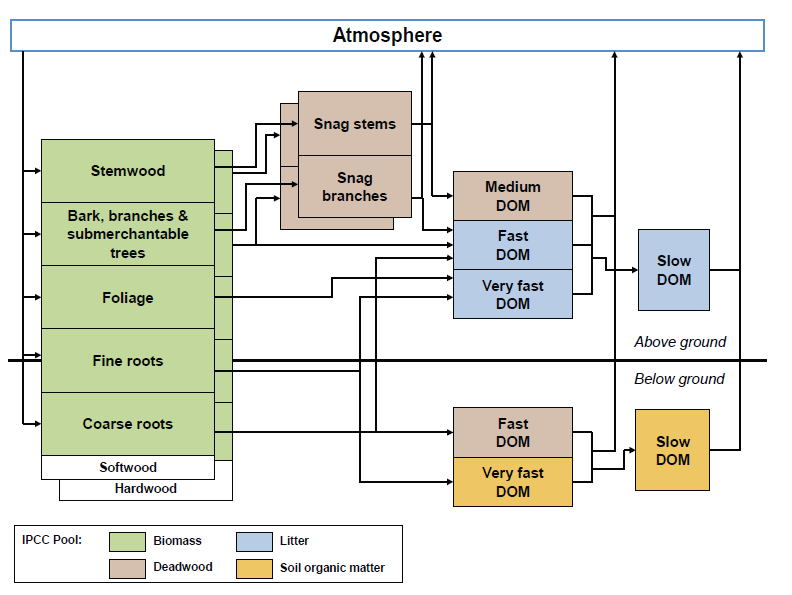


Figure S1 Carbon pools and transfers simulated by the CBM-CFS3. Source: Figure A3-12, Environment Canada (16). The aboveground/belowground line indicates the separation between the soil organic horizon and the mineral soil.

Disturbances trigger different combinations of C transfers, based on the disturbance type and severity, the forest type affected and the ecological region. The impact of a disturbance is defined by a disturbance matrix, which specifies the proportion of C in each ecosystem pool that is transferred to other pools, released to the atmosphere (in different GHGs) or transferred to Harvested Wood Products.  Figure S2 shows a matrix, simulating clear-cut harvesting and salvage logging with an 85% utilization rate.


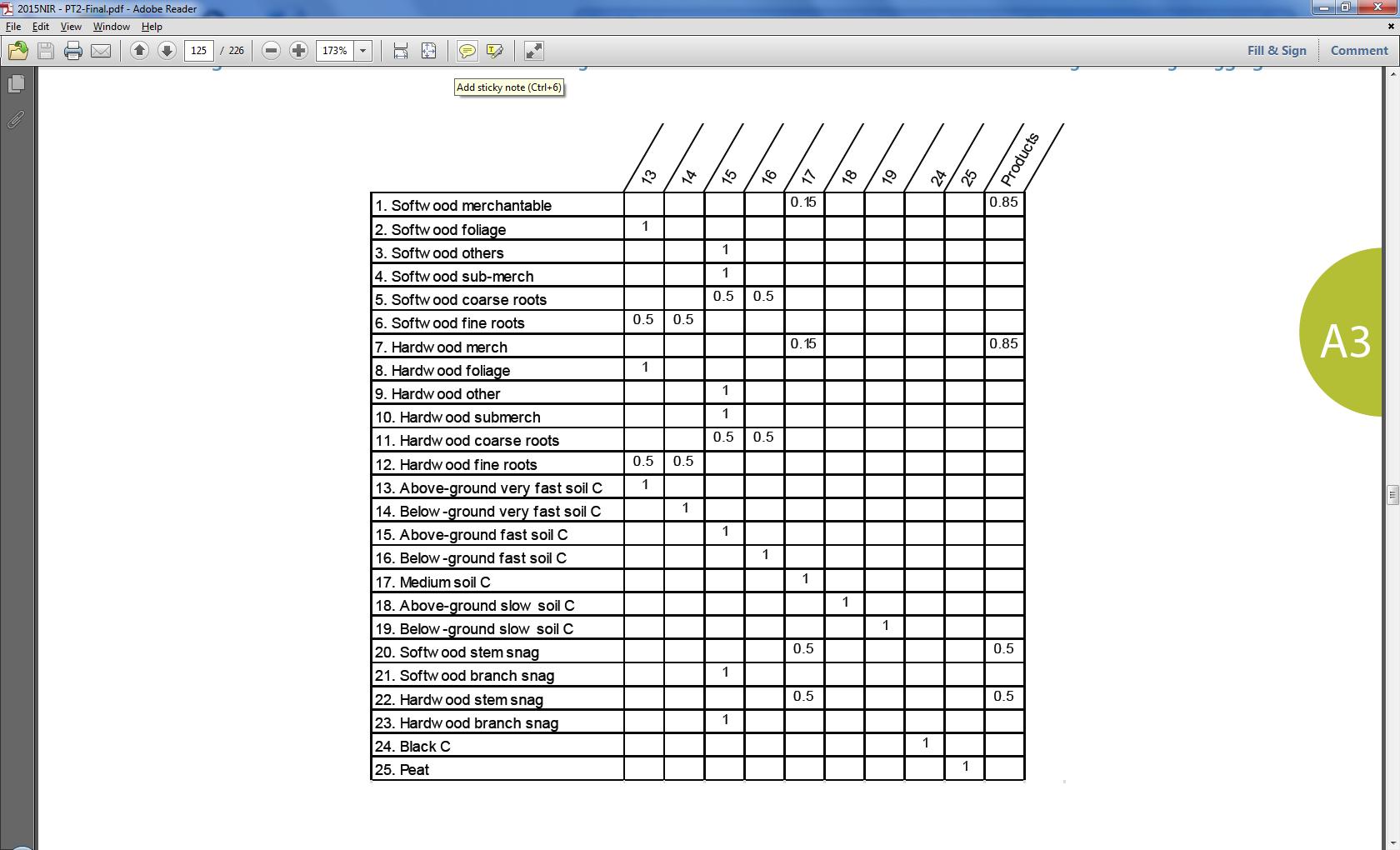


Figure S2 Disturbance matrix simulating the C transfer associated with a clearcut and salvage harvest. Source: Figure A3-13, Environment Canada (16).

Annual harvest for each harvest method (clearcut, thinning, etc) includes (1) eligibility criteria to exclude certain stands from harvesting (e.g., stands identified in the inventory as having been set aside as reserves or immature trees); (2) sorting routines to sort eligible stands according to harvest priority; and (3) harvesting stands in order of descending priority until the annual harvest C or area target has been achieved. A harvesting efficiency parameter can be used to set aside a proportion of the stand for fine-tuning stand selection. Harvesting efficiency is defined as the proportion of any record that is allowed to be harvested in any year, thus forcing harvest selections deeper into priority lists to avoid high grading and to emulate access limitations encountered during operational harvest scheduling.

# S2. Harvested Wood Product Tracking

Carbon that was transferred out of the forest ecosystem due to harvesting was modeled by the Carbon Budget Model Framework for Harvested Wood Products (CBM-FHWP) – which tracks the fate of harvested C throughout the lifetime of harvest wood products (HWP) including bioenergy and post-consumer treatment. CBM-FHWP tracks the C flow associated with HWPs manufactured from wood that was harvested within the forest management unit (FMU) regardless of where in the world these products reside – the IPCC Production Approach for estimation of HWP C balances (17). This framework is part of Canada’s annual GHG National Inventory Report (3). Sawnwood and other industrial roundwood are assumed to have a 35 year half-life, panels have a 25 year half-life, and pulp and paper have a 2 year half-life (17).

Discarded products are included in the framework and can be used for energy and/or sent to landfills. A portion of solid wood and paper products sent to landfills are degradable, with half‑lives that vary by region.

# S3. Bioenergy from harvest residues

Displaced emissions are defined as emissions that would have occurred if *Base Case* energy sources had been used. Displaced emissions were estimated two ways: (1) using previously published methods briefly described below, but detailed information can be found in Smyth et al. (18, 19), and (1) refining the displacement factors by including community-level energy demand and fuel mix in the BC case study (20), and by transporting residues across the FMU boundary to the adjacent community of Thunder Bay in the ON case study.

## S3.1 Regional displaced emissions

Displaced emissions were estimated for the Cranbrook and Dog River FMU by multiplying the captured harvest residues by a regionally-determined displacement factor. The displacement factor was estimated for each region by selecting the type, size and number of bioenergy facilities that maximized displaced emissions based on the (1) available harvest residues, (2) regional energy consumption, and (3) *Base Case* fuel mix. *Base Case* energy sources for electricity were from projected energy sources (Table S3) for each province/territory (21) and sources of heat were from contemporary (2012) energy sources (22) because projections were not available. For FMUs that contained a remote community, such as Dog River, the regional fuel mix was estimated from the provincial or territorial average and then adjusted to include the contemporary remote community fuel mix using to a weighted-proportion of the population of the remote community and regional population (Natural Resources Canada Remote Communities Database, 2014). Remote communities are defined as those that are not connected to an electricity grid and that therefore have a different fuel mix than the jurisdictional-average fuel mix.

Energy consumption for heat and electricity was estimated from each jurisdiction’s per capita energy use and contemporary population estimates from census data (24).

We assumed that all captured harvest residues were first used within their FMU to produce heat and electricity to meet local demand, which was estimated from per capita use multiplied by the population within the FMU. Heat production was constrained to local demand, and any excess harvest residues (beyond that needed for local demand) were consumed to generate grid-electricity which was assumed to displace the average electricity fuel mix.

The nine bioenergy facilities included three different types of facilities (heat, power, and combined heat and power) and three different sizes of facilities, ranging from 200 kW turbines to a 10 MWh steam cycle power facility, Table S4.

## S3.2 Refined displaced emissions

The refined set of displacement factors estimated displacement factors for each of the five communities within the Cranbrook FMU, based on their share of harvest residues estimated from the cheapest transportation route from cutblock to community. Energy demands were lower than the FMU average, and the average displacement factor was found to have a lower average value of 0.46 for the five communities, with a range of 0.23 to 0.66. For Dog River, we refined the base case energy demand to exclude coke and petcoke in heating fuels because Ontario has phased out coal usage. We also transported the harvest residues to the nearby community of Thunder Bay, which significantly increased the displacement factor from 0.38 to 1.0 because of the substitution benefits of avoiding heating fossil fuels

| Table S1. Regional per capita energy consumption and energy fuel mix for projected electricity production (E) and heat production (H). a) FMU level consumption. b) Community level consumption. Percentages have been rounded, and may not add to 100%. | | | | | | | | | | | | | | |  |
| --- | --- | --- | --- | --- | --- | --- | --- | --- | --- | --- | --- | --- | --- | --- | --- |
| Region | E: consumed | H: consumed | H: NG | H: Electricity | H: Fuel Oil | H: Propane | H: Wood | H: Coke Petcoke | E: Coal | E: Fuel Oil | E: Diesel | E: NG^a^ | E: rest^b^ | Average Electricity grid emissions | |
| Region | (MWh per person) | (MWh per person) | (%) | (%) | (%) | (%) | (%) | (%) | (%) | (%) | (%) | (%) | (%) | (kg  CO_2_e MWh^-1^) | |
| a) Cranbrook FMU | 28 | 56 | 42 | 8 | 9 | 0 | 35 | 5 | 0 | 0 | 0 | 13 | 87 | 58 | |
| Dog River FMU | 13 | 25.9 | 57 | 4 | 5 | 0 | 8 | 26 | 0 | 0 | 66 | 7 | 27 | 88 | |
| b) Community level |  |  |  |  |  |  |  |  |  |  |  |  |  |  | |
| Thunder Bay | 13 | 25.9 | 77 | 6 | 7 | 0 | 11 | 0 | 0 | 0 | 0 | 19 | 81 | 88 | |
| Cranbrook | 28 | 14.9 | 89 | 0 | 2 | 3 | 7 | 0 | 0 | 0 | 0 | 13 | 87 | 58 | |
| Elkford | 28 | 20.1 | 76 | 0 | 4 | 6 | 14 | 0 | 0 | 0 | 0 | 13 | 87 | 58 | |
| Fernie | 28 | 23.1 | 91 | 0 | 1 | 2 | 5 | 0 | 0 | 0 | 0 | 13 | 87 | 58 | |
| Kimberley | 28 | 19.9 | 80 | 0 | 3 | 5 | 12 | 0 | 0 | 0 | 0 | 13 | 87 | 58 | |
| Sparwood | 28 | 19.4 | 84 | 0 | 2 | 4 | 10 | 0 | 0 | 0 | 0 | 13 | 87 | 58 | |
|  |  |  |  |  |  |  |  |  |  |  |  |  |  |  |  |
| ^a^ Natural Gas (NG) |  |  |  |  |  |  |  |  |  |  |  |  |  |  |  |
| ^b^ “rest” includes power generation from hydro-electricity, wind and tide, biomass and uranium. | | | | | | | |  |  |  |  |  |  |  |  |

Table S2. Description of the three sizes (small, medium, large) of three types (district heat, power and combined heat and power) for the nine selected bioenergy facilities. Assuming 340 operating days, 24 hr per day operating hours and a wood energy content of 20 GJ odt^-1^.

| Facility Type | Facility Description | Biomass demand  (kodt yr^-1^) | Electrical conversion  rate  (MWh/odt) | Thermal conversion  rate  (GJ/odt) | Assumed electrical efficiency  (%) | Assumed thermal efficiency  (%) | Implied  overall efficiency  (%) |
| --- | --- | --- | --- | --- | --- | --- | --- |
| Heat | 0.4 MWth boiler for district heating^a^ | 0.783 | - | 15.0 | - | 75 | 75 |
|  | 2.3 MWth boiler for district heating^b^ | 3.97 | - | 17.0 | - | 85 | 85 |
|  | 6.62 MWth process heat via syngas^c^ | 11.58 | - | 16.8 | - | 84 | 84 |
| Power | 0.2 MWe gas turbine^d^ | 1.60 | 1.02 | - | 18 | - | 18 |
|  | 5 MWe steam cycle^c^ | 34.97 | 1.17 | - | 21 | - | 21 |
|  | 10 MWe steam cycle^c^ | 63.86 | 1.28 | - | 23 | - | 23 |
| CHP | 0.2 MWe, 0.98 MWth Organic Rankine Cycle^e^ | 2.09 | 0.78 | 14.0 | 14 | 70 | 84 |
|  | 1.8 MWe and 4.5MWth steam turbine^f^ | 10.58 | 1.39 | 10.8 | 25 | 54 | 79 |
|  | 8 MWe CHP steam turbine^c^ | 46.87 | 1.39 | 5.88 | 25 | 29 | 54 |

^a^ RETScreen International (2015)

^b^ RETScreen International (2015)

^c^ Biopathways (FPAC & FPInnovations, 2011)

^d^ Arena et al. (2010)

^e^ Wood and Rowley (2011)

^f^ Pröll et al. (2011)

Table S3. Bioenergy facility selection for a) Dog River and Cranbrook FMUs, b) community of Thunder Bay ON and communities within the Cranbrook FMU. Medium and Large electricity facilities (not shown) were not selected. Abbreviations: CHP *Combined Heat and Power,* H *Heat*, E *Electricity*.

|  | Region | CHP Small | CHP Med. | CHP Large | H: Small | H: Med. | H: Large | E: Small | Total Avoided Emission (tCO2e) | Displacement Factor | Local Electricity Intensity (kgCO2e MWh-1) |
| --- | --- | --- | --- | --- | --- | --- | --- | --- | --- | --- | --- |
| a | Dog River FMU, ON | 1 |  |  | 1 |  |  | 18 | 22120 | 0.38 | 559 |
|  | Cranbrook FMU, BC | 2 | 11 |  | 2 | 7 | 1 |  | 280747 | 0.95 | 58 |
| b | Thunder Bay, ON | 3 | 2 |  |  | 1 |  |  | 58000 | 1.00 | 88 |
|  | Cranbrook | 2 | 3 |  |  | 5 | 1 |  | 71331 | 0.57 | 14 |
|  | Elkford |  |  |  | 1 | 2 |  |  | 10755 | 0.62 | 14 |
|  | Fernie |  |  | 1 | 1 | 1 |  | 5 | 25831 | 0.23 | 14 |
|  | Kimberley | 1 |  |  |  | 5 |  |  | 26728 | 0.66 | 14 |
|  | Sparwood |  |  |  | 4 |  |  |  | 3672 | 0.58 | 14 |

# S4. 2030 Cumulative mitigation results


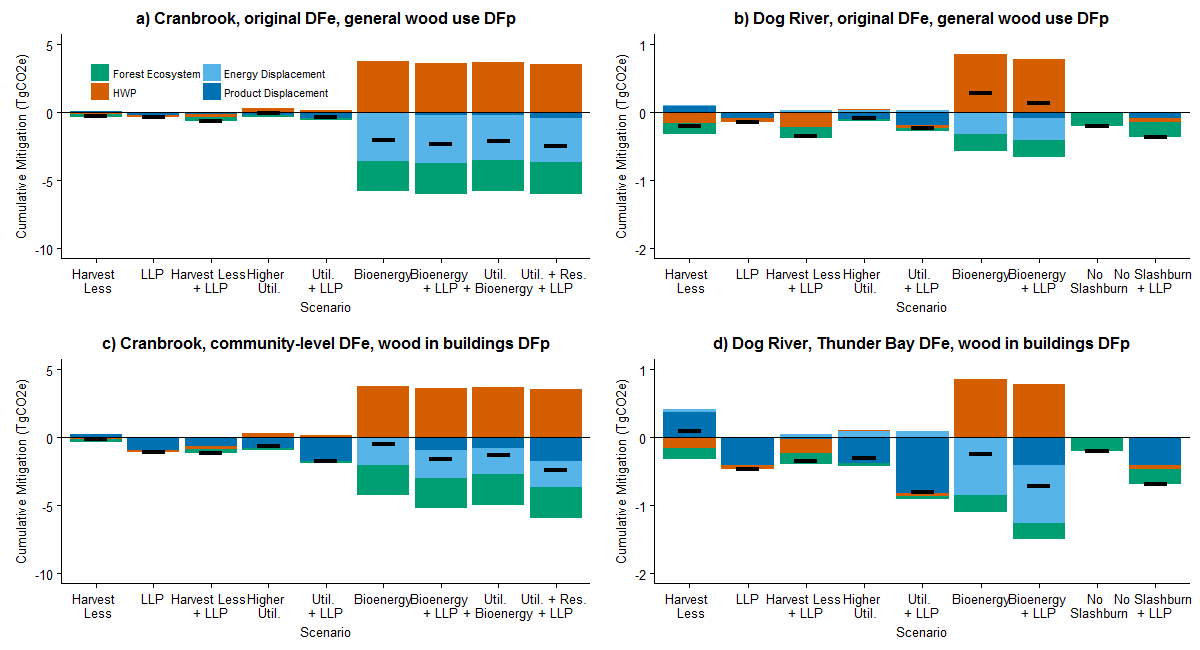


Figure S3 Total and component cumulative mitigation in 2030 for Cranbrook and Dog River FMUs with (a and b) displacement factors (DF) based on FMU-level energy substitution (DFe) and broad end-uses for solid wood products (DFp) or (c and d) displacement factors based on community-level energy substitution and incremental solid wood products for use in building construction. The black horizontal line shows the total mitigation. Abbreviations: LLP *Longer Lived Products,* Higher Util. or Util. *Higher Utilization,* Bioenergy or Res. *Harvest Residues for Bioenergy.*

References

1. Kurz WA, Dymond CC, White TM, Stinson G, Shaw CH, Rampley GJ, et al. CBM-CFS3: A model of carbon-dynamics in forestry and land-use change implementing IPCC standards. Ecol Model. 2009;220(4):480-504.

2. Kurz WA, Apps M, Banfield E, Stinson G. Forest carbon accounting at the operational scale. Forestry Chronicle. 2002;78(5):672-9.

3. Environment and Climate Change Canada. National Inventory Report: 1990-2015, greenhouse gas sources and sinks in Canada Ottawa, ON: Environment and Climate Change Canada, Greenhouse Gas Division; 2017 2017.

4. Stinson G, Kurz WA, Smyth CE, Neilson ET, Dymond CC, Metsaranta JM, et al. An inventory-based analysis of Canada's managed forest carbon dynamics, 1990 to 2008. Global change Biol. 2011;17(6):2227-44.

5. Kim M, Lee WK, Kurz WA, Kwak DA, Morken S, Smyth CE, et al. Estimating carbon dynamics in forest carbon pools under IPCC standards in South Korea using CBM-CFS3. iForest - Biogeosciences and Forestry. 2016:e1-e10.

6. Pilli R, Grassi G, Kurz WA, Moris JV, Viñas RA. Modelling forest carbon stock changes as affected by harvest and natural disturbances. II. EU-level analysis. Carbon Balance and Management. 2016;11(1):1-19.

7. Boudewyn P, Song X, Magnussen S, Gillis MD. Model-based, volume-to-biomass conversion for forested and vegetated land in Canada. Information Report. 2007;BC-X-411.

8. Li Z, Kurz WA, Apps MJ, Beukema SJ. Belowground biomass dynamics in the Carbon Budget Model of the Canadian Forest Sector: recent improvements and implications for the estimation of NPP and NEP. Can J For Res. 2003;33(1):126-36.

9. Shaw CH, Hilger AB, Metsaranta J, Kurz WA, Russo G, Eichel F, et al. Evaluation of simulated estimates of forest ecosystem carbon stocks using ground plot data from Canada's National Forest Inventory. Ecol Model. 2014;272(0):323-47.

10. Smyth CE, Trofymow JA, Kurz WA, CIDET Working Group. Decreasing uncertainty in CBM-CFS3 estimates of forest soil C sources and sinks through use of long-term data from the Canadian Intersite Decomposition Experiment. Victoria: Natural Resources Canada, Canadian Forest Service, Pacific Forestry Centre; 2010. Report No.: BC Information Report BC-X-422

11. Smyth CE, Kurz WA, Trofymow JA. Including the effects of water stress on decomposition in the Carbon Budget Model of the Canadian Forest Sector CBM-CFS3. Ecol Model. 2011;222(5):1080-91.

12. White T, Luckai N, Larocque GR, Kurz WA, Smyth C. A practical approach for assessing the sensitivity of the Carbon Budget Model of the Canadian Forest Sector (CBM-CFS3). Ecol Model. 2008;219(3-4):373-82.

13. Smyth CE, Kurz WA. Forest soil decomposition and its contribution to heterotrophic respiration: A case study based on Canada. Soil Biology and Biochemistry. 2013;67(0):155-65.

14. Smyth CE, Kurz WA, Neilson ET, Stinson G. National-scale estimates of forest root biomass carbon stocks and associated carbon fluxes in Canada. Global Biogeochem Cycles. 2013;27(4):1262-73.

15. Metsaranta JM, Shaw CH, Kurz WA, Boisvenue C, Morken S. Uncertainty of inventory-based estimates of the carbon dynamics of Canada’s managed forest (1990-2014). Can J For Res. 2017;In Press.

16. Environment Canada. National Inventory Report: 1990-2013, greenhouse gas sources and sinks in Canada Ottawa, ON: Environment Canada, Greenhouse Gas Division; 2015 2015.

17. IPCC. Revised supplementary methods and good practice guidance arising from the Kyoto Protocol. Kanagawa, Japan: Institute for Global Environmental Strategies; 2013.

18. Smyth CE, Rampley GJ, Lemprière TC, Schwab O, Kurz WA. Estimating product and energy substitution benefits in national-scale mitigation analyses for Canada. Global Change Biology Bioenergy. 2017b;9:1071–84.

19. Smyth C, Kurz WA, Rampley GJ, Lemprière TC, Schwab O. Climate change mitigation potential of local use of harvest residues for bioenergy in Canada. Global Change Biology Bioenergy. 2017a;9:817–32.

20. Community Energy and Emissions Inventory [Internet]. 2016 [cited Nov 2, 2016]. Available from: http://www2.gov.bc.ca/gov/content/environment/climate-change/data/ceei.

21. National Energy Board. Canada’s Energy Future 2013 - Energy Supply and Demand Projections to 2035 - Appendices. https://www.neb-one.gc.ca/nrg/ntgrtd/ftr/2013/ppndcs/ppndcs-eng.html; 2013.

22. Comprehensive Energy Use Database [Internet]. 2015. Available from: http://oee.nrcan.gc.ca/corporate/statistics/neud/dpa/menus/trends/comprehensive_tables/list.cfm.

23. Remote Communities Database [Internet]. 2015. Available from: http://www2.nrcan.gc.ca/eneene/sources/rcd-bce/index.cfm?fuseaction=admin.home1.

24. 2011. Dissemination Block boundary file.
